# Online biophysical predictions for SARS-CoV-2 proteins

**DOI:** 10.1186/s12860-021-00362-w

**Published:** 2021-04-23

**Authors:** Luciano Kagami, Joel Roca-Martínez, Jose Gavaldá-García, Pathmanaban Ramasamy, K. Anton Feenstra, Wim F. Vranken

**Affiliations:** 1 Interuniversity Institute of Bioinformatics in Brussels, ULB-VUB, Triomflaan, 1050 Brussels, Belgium; 2 Structural Biology Brussels, Vrije Universiteit Brussel, Pleinlaan 2, 1050 Brussels, Belgium; 3 VIB Structural Biology Research Centre, Pleinlaan 2, 1050 Brussels, Belgium; 4 VIB-UGent Center for Medical Biotechnology, VIB, 9000 Ghent, Belgium; 5 Department of Biomolecular Medicine, Faculty of Health Sciences and Medicine, Ghent University, 9000 Ghent, Belgium; 6 IBIVU – Center for Integrative Bioinformatics, Department of Computer Science, Vrije Universiteit Amsterdam, Amsterdam, 1081HV The Netherlands; 7 AIMMS – Amsterdam Institute for Molecules Medicines and Systems, Vrije Universiteit Amsterdam, Amsterdam, 1081HV The Netherlands

**Keywords:** Proteins, Single sequence based predictions, Biophysical features, SARS-CoV-2, COVID-19

## Abstract

**Background:**

The SARS-CoV-2 virus, the causative agent of COVID-19, consists of an assembly of proteins that determine its infectious and immunological behavior, as well as its response to therapeutics. Major structural biology efforts on these proteins have already provided essential insights into the mode of action of the virus, as well as avenues for structure-based drug design. However, not all of the SARS-CoV-2 proteins, or regions thereof, have a well-defined three-dimensional structure, and as such might exhibit ambiguous, dynamic behaviour that is not evident from static structure representations, nor from molecular dynamics simulations using these structures.

**Main:**

We present a website (https://bio2byte.be/sars2/) that provides protein sequence-based predictions of the backbone and side-chain dynamics and conformational propensities of these proteins, as well as derived early folding, disorder, β-sheet aggregation, protein-protein interaction and epitope propensities. These predictions attempt to capture the inherent biophysical propensities encoded in the sequence, rather than context-dependent behaviour such as the final folded state. In addition, we provide the biophysical variation that is observed in homologous proteins, which gives an indication of the limits of their functionally relevant biophysical behaviour.

**Conclusion:**

The https://bio2byte.be/sars2/ website provides a range of protein sequence-based predictions for 27 SARS-CoV-2 proteins, enabling researchers to form hypotheses about their possible functional modes of action.

**Supplementary Information:**

The online version contains supplementary material available at 10.1186/s12860-021-00362-w.

## Background

The SARS-CoV-2 virus, the causative agent of COVID-19, consists of an assembly of proteins that determine its infectious and immunological behavior, as well as its response to therapeutics. Major structural biology efforts on these proteins have already provided essential insights into the mode of action of the virus, as well as avenues for structure-based drug design [1] . However, not all of the SARS-CoV-2 proteins, or regions thereof, have a well-defined three-dimensional structure, and as such might exhibit ambiguous, dynamic behaviour that is not evident from static structure representations generated by structural biology approaches, nor from molecular dynamics simulations using these structures.

**Fig. 1:**
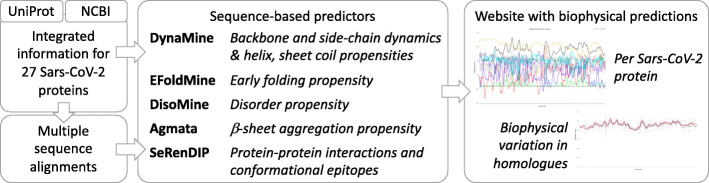
Overview of the employed workflow and the incorporated predictions

We here present a website [2] that provides extensive protein sequence-based predictions for the SARS-CoV-2 proteins, which can help to pinpoint previously unidentified behavior or features of these proteins that might not be captured by structural biology or molecular dynamics approaches. The predictions include the DynaMine backbone [3, 4] and side-chain dynamics [5] as well as conformational propensities [5], and derived DisoMine disorder [6], EFoldMine early folding [5], Agmata β-sheet aggregation [7], SeRenDIP protein-protein interaction [8] and SeRenDIP-CE conformational epitope propensities [9]. These predictions attempt to capture the ‘emergent’ properties of the proteins, so the inherent biophysical propensities encoded in the sequence, rather than context-dependent behaviour such as the final folded state. This approach has already shown promise in, for example, detecting remote homologues by biophysical similarity, which gives more accurate results than directly using amino acid information [10]. We apply this concept on the SARS-CoV-2 proteins by incorporating evolutionary information, so enabling us to display the biophysical variation observed in homologous proteins, which indicates likely limits of their functionally relevant biophysical behaviour (Fig. 1). The information we provide is not directly applicable in, for example, drug design against SARS-CoV-2 proteins, but might help explain their mode of action if they act against regions for which we have no direct structural knowledge. The aim of our website is therefore to provide leads for researchers with regard to the possible mode of action of these proteins.

## Construction and content

### Datasets

The target amino acid sequences of the 27 proteins were obtained by integrating information from the UniProt [11] COVID-19 section, after filtering on ‘Other organisms’ by ‘Severe acute respiratory syndrome coronavirus 2’, and the NCBI Sars-CoV-2 website (https://www.ncbi.nlm.nih.gov/sars-cov-2/). The full UniProt P0DTC1 and P0DTD1 entries were excluded from this list, as they are spliced into components included in our list. Multiple sequence alignments (MSAs) for these sequences were obtained using the following steps:

1. MSAs for homologous sequences were obtained using the UniProt BLAST tool against the UniRef90 database, using default parameters and limiting the number of hits to 250.
2. CD-HIT was applied to each MSA using sequence identity threshold of 70%, except for P0DTC2, where an 80% threshold was used to avoid discarding too many sequences.
3. The representative sequences found by CD-HIT, with the target sequences added if necessary, were aligned using the Clustal Omega based online UniProt Alignment tool.
4. C- and N-terminal regions from homologous proteins extending beyond the target sequence were removed.

Note that for the 15 non-structural proteins (Nsp), the full ORF1ab protein sequence was used for the BLAST search, CD-HIT and alignment (steps 1–3). It was then split into the component Nsps.

### Predictions

On each target sequence, the backbone dynamics (DynaMine) [3, 4], and related side-chain dynamics and conformational propensities [5] were predicted at the per-amino acid level. These methods are based on the per-residue characteristics (e.g. backbone dynamics) derived from NMR chemical shift values, and use a linear regression model for the prediction. Early folding probabilities per residue were predicted using EFoldMine [5], which uses as input features the five previously mentioned DynaMine values for a 5-residue fragment, resulting in a 25 dimensional feature vector that was trained using a Support Vector Machine (SVM) on a set of high-resolution per-residue hydrogen-deuterium exchange (HDX) data from NMR experiments for 30 proteins. Disorder propensities were calculated with DisoMine [6], which uses a Recurrent Neural Network (RNN) trained on data of 535 non-redundant proteins from DisProt [12], with as input features the DynaMine backbone and side-chain dynamics values, the EFoldMine values, and PSIPRED secondary structure predictions [13]. β-sheet aggregation was predicted with Agmata [7], which uses logistic regression on the previously mentioned 5 DynaMine features on a 3-residue window in a statistical potential model to pinpoint residues that could lead to β-sheet aggregation. Protein-protein interactions (SeRenDIP) [8, 14] were predicted using a random-forest (RF) model trained on a mixed homo- and heteromeric PPI dataset derived from the PDB [15] . Epitope propensities were predicted with SeRenDIP-CE [9], which uses an RF model trained on a dataset of antigen sequences annotated with antibody-binding regions derived from SabDab [9, 16] . Predictions of FUS-like phase separation were performed with PSPer [17], which employs an HMM-like model to assign domains (e.g. low complexity region) to a protein sequence, and assesses whether these domains can combine to create FUS-like phase separation behavior. All predictions, except for Agmata, SeRenDIP, SeRenDIP-CE and PSPer, were then run on all individual sequences in the MSAs, with the values mapped back to the MSA, so obtaining per MSA column a list of prediction values. A standard box plot approach was then applied to each per-column list of values to identify the median, first and third quartile, and outlier range per biophysical feature per column in each MSA.

### Phosphorylation sites

Experimentally validated phosphorylation sites were obtained from two SARS-CoV-2 phosphoproteome projects (PXD020183, PXD019113) in the PRIDE repository [18]. The search files for the projects were downloaded and processed to extract the phospho-site information. Since the data processing protocol varies between the projects depending on the search engine used, we only considered the phosphor-sites that are seen in more than one project with a localization probability of > 0.6.

### Website

The information is visualized online using the Django framework, with the ApexCharts JavaScript library employed for visualization of the predictions and their MSA distribution.

## Utility and discussion

### Website description

The home page provides a brief statement on the purpose of the website, and how to proceed. On the ‘Entries’ page (available from the top bar), the 27 available SARS-CoV-2 proteins are listed in a sortable table containing their ORF Name, NCBI RefSeq ID, UniProt ID, sequence length and protein category. For each entry, accessed by clicking on the ORF Name or any of the other identifiers, the following information is provided on the left-hand side:

- Link to structure(s) in the PDB [19] via the PDBe-KB [20] (if available)
- Link to UniProt information (if available)
- Link to NCBI information (if available)
- PSPer predictions about the possible phase-separation behavior of this protein (if available, only for proteins of length 130 or more)
- Download all predictions for this protein in JSON format
- Download of the multiple sequence alignment (MSA) for this protein

The top plot of the per-protein pages visualises all incorporated predictions (y-axis) in function of the protein sequence (x-axis). Hovering with the pointer over the graph will display the residue number (below the x-axis) and the corresponding prediction values (in the legend). The sequence-based predictions are listed in Table 1, and reflect ‘emerging’ properties, so what the sequence is capable of, not necessarily what it will adopt in a final fold. Each prediction can be toggled on/off by clicking on the corresponding name in the legend of the plot. All predictors, due to their underlying methodology, generate single values per residue without providing an uncertainty range, which limits their interpretability. The use of sequence information from homologous proteins (see next paragraph) partially addresses this issue by incorporating information about the likely variation of the predicted parameters in evolution.

**Table 1:** Overview of the prediction software used

Software	Type	Color	Description
DynaMine	**Backbone dynamics**	Black	Rigidity of the backbone, higher values mean backbone movements are more likely to be limited; values > 1.0 indicate membrane spanning regions, > 0.8 rigid conformations, < 0.69 flexible regions. Residues with 0.80–0.69 values are ‘context’ dependent and capable of being either rigid or flexible.
	**Sidechain dynamics**	Grey	Rigidity of the sidechain, higher values mean the sidechain is more likely to be conformationally restricted. These values are highly dependent on the amino acid type (i.e. a Trp will be rigid, an Asp flexible).
	**Sheet, helix, coil propen-sities**	Blue, red, purple	The propensity of the residue, based on local amino acid context, to adopt helix, sheet or coil conformations. Higher values indicate higher propensities.
EFoldMine	**Early folding propensity**	Green	Likelihood that this residue will adopt a persistent conformation based on only local interactions with other amino acids. Values > 0.169 indicate residues most likely to start the protein folding process.
DisoMine	**Disorder**	Yellow	Likelihood that this residue will be disordered (highly dynamic, many conformations). Values > 0.5 indicate that this is most likely a disordered residue.
Agmata	**Aggregation propensity**	Dark green	Residues with higher values indicate residues likely to be involved in β-sheet aggregation. The values displayed in the plots are divided by a factor 20 from the original.
SeRenDIP	**Protein-protein interactions**	Cyan	Indicates residues likely to participate in protein-protein interactions (PPIs). Values > 0.5 indicate residues that are most likely involved in PPIs.
SeRenDIP-CE	**Conformat-ional epitope regions**	Sea-green	Indicates residues likely to be part of a conformational (discontinuous) epitope (CE) region. Values > 0.5 indicate residues that are most likely involved in CEs.

The second plot visualises the MSA-based variation of a specific predicted feature (like backbone dynamics) for single-sequence based predictions, again with the prediction value (y-axis) in function of the protein sequence (x-axis). The type of prediction shown can be selected using the ‘Select prediction’ selection box, with the plot showing median (black), first and third quartile (dark grey) and outlier range (light grey) of the distribution per column in the MSA, as well as the original prediction for the target protein itself (red), which corresponds to the prediction in the top graph. These distributions reflect the ‘evolutionary allowed’ range of the biophysical features, which as we have previously shown tends to be only weakly correlated with amino acid sequence-based MSA measures such as entropy [8, 21]. Values of the red line outside of the quartile range therefore indicate rather unusual behaviour for this particular protein compared to its homologues, and might indicate interesting areas where this SARS-CoV-2 protein differs from other proteins. Finally, at the bottom of the page links are provided to the PSPer predictions and the JSON with all the prediction values, as well as their distributions, for this protein. Note that only MSA columns for which there is no gap in the target sequence are shown.

The predictions we provide are limited in the sense that they provide a single per-residue value that in itself does not give detailed information on the overall protein behavior. However, when these values are considered in relation to each other, or contextualised in relation to external information such as structural data, these values can give useful pointers to possible behaviors of the proteins (or regions thereof) for which we do not yet have much information, as illustrated in the next section through a use-case example. The aim of our website is therefore not to give definitive answers in relation to the behavior of SARS-CoV-2 proteins, but rather to enable researchers to explore which possible behavior these proteins (or regions thereof) might have (e.g. aggregation tendency), so providing leads on their possible mode of action.

### Use-case example

**Fig. 2:**
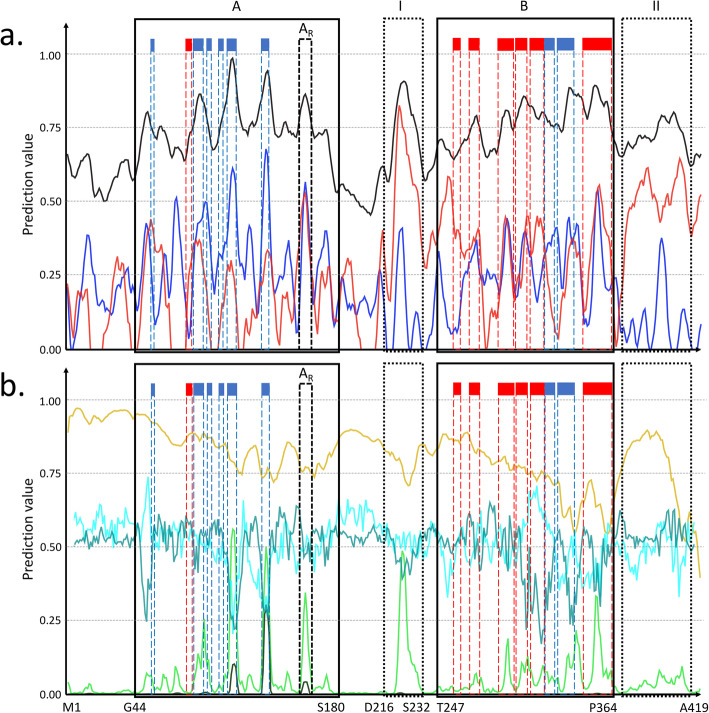
Predictions for the P0DTC9 SARS-CoV-2 protein amino acids (x-axis) for **a**) backbone dynamics (black), helix (red) and sheet (blue) propensity, and **b**) early folding (light green), disorder (yellow) β-sheet aggregation (dark green), protein interaction (cyan) and epitopes (seagreen). The two regions for which structures have been determined are indicated by black boxes (**a**, **b**), with annotations for consensus α-helix (red boxes) and β-strands (blue boxes) based on these structures included. Regional highlights not evident in these structures (A_R_, I, II) are discussed in the text. On the interactive plots on the server, predictions can be toggled on and off by clicking on their name

The UniProt P0DTC9 protein (RefSeq ID YP_009724397) is a nucleoprotein of 419 amino acids with both monomeric and oligomeric forms that interact with RNA, as well as with protein M and NSP3. These interactions tether the genome to the newly translated replicase-transcriptase complex at a very early stage of infection. Structural information is available for UniProt-numbered residues Gly44-Ser180 (Fig. 2, box A, based on PDB codes 6yi3, 7act, 7acs), which mediates RNA binding, and for Thr247-Pro364 (Fig. 2, box B, based on PDB code 6zco), which are involved in oligomerisation (see also [22]). The predictions for this protein, displayed in Fig. 2 (for an interactive version, please see [23]), show a high propensity for disorder throughout the protein, with backbone dynamics also indicating overall high flexibility (values below 0.69), except for the previously mentioned Gly44-Ser180 and Thr247-Pro364 regions, which have been observed to fold. The N-terminal region prior to Gly44 is highly flexible, with some helix and sheet propensity, and a propensity for protein-protein interactions, but with no indications of early folding or aggregation. It also contains multiple confirmed phosphorylation sites (Ser23 and Ser26), hinting at a possible regulation role.

For the first folded domain (box A), the regions confirmed by PBDe-KB to form α-helices (red dotted boxes) and β-strands (blue dotted boxes) are indicated, which tend to correspond to rigid areas with strong secondary structure propensity. Interestingly, the A_R_ region from Asn153 to Gln 163 (black box) does not have a regular secondary structure, but corresponds to an extended region that loops over the outside of the protein. Given the very high prediction values for rigidity, helix and sheet propensity (equal) and early folding, combined with a peak in aggregation, this region could have an important role in the folding process and overall behavior of this protein, even though it does not particularly stand out in the solved crystal structures. There is also notable aggregation tendency corresponding to the 5th and 6th β-strands, and a high epitope propensity in the subsequent region between the 6th β-strand and the A_R_ region. A confirmed phosphorylation site is Ser79, at the beginning of the first α-helix.

The subsequent region between Ser180 and Thr247 contains a region with relatively consistent properties from Ser180-Gly215, indicating a highly flexible linker that connects the two structured regions, but with an interestingly elevated PPI propensity. This area also contains multiple confirmed phosphorylation sites (Ser187, Ser194, Ser197, Ser201, Ser206), indicating a regulatory role. The region from Asp216-Thr247 (box I), on the other hand, shows strong peaks in both backbone rigidity and helical propensity from Asp216-Ser232, with indications that this region is prone to early folding. To the best of our knowledge, no structural or functional information is available for this region, but the predictions again indicate that this area could well play a role in regulation, for example, by blocking a site when this helix is formed, or by constraining the distance between the two domains by adapting the overall linker length. Noteworthy is also that both the backbone dynamics and helical propensity, but especially the early folding, are above the third quartile range observed in homologous proteins (Additional file 1; Fig. S1, S2), indicating that this region has a stronger tendency to autonomously form a helix in the SARS-CoV-2 protein compared to its close homologues.

The oligomerisation domain (box B) shows a strong epitope propensity from Ala273-Asn285, and a very strong PPI propensity from Lys299-Met322, corresponding to the 5th and 6th α-helices (Lys299-Ile304 and Gln306-Gly316) and 7th β-strand (Met317-Met322), in line with orientation in the dimer where β-strands 7 and 8 (Val324-Tyr333) form a four-stranded sheet with the corresponding strands from the other monomer, with α-helix 6 below the sheet and also part of the homodimer interface [24]. The conformational preference for helix formation is already indicated by the predictions, as are the two β-strand regions.

Finally, the C-terminal region after Pro364 (box II) is in the ‘context-dependent’ zone of the backbone dynamics predictions between 0.80 and 0.69, indicating it could fold, in this case likely into a helix as it also has a strong helical propensity. This again indicates a possible regulatory or transient binding role, possibly to a protein as it has peaks of fairly high PPI propensity. There are also high peaks of epitope propensity in this region, particularly around Pro365 and Leu395-Gln408. The region also contains multiple likely phosphorylation sites.

## Conclusions

This website provides researchers with information on possible behaviours of SARS-CoV-2 proteins that are not evident from the static models generated by structural biology, nor from molecular dynamics simulations based on these models. It enables the exploration of these proteins from a different perspective and should help further our understanding of the mode of action of the overall virus.

## Supplementary Information

**Additional file 1: Supplementary data.** Contains two additional Figs. (S1, S2) showing the spread of biophysical predictions based on the multiple sequence alignment (MSA) for the P0DTC9 SARS-CoV-2.

## Data Availability

All data are freely available as JSON or FASTA files via per-protein links in the http://sars2.bio2byte.be/ website, and will remain available for download.
